# Effects of pretreatment with medetomidine, midazolam, ketamine, and their combinations on stress-related hormonal and metabolic responses in isoflurane-anesthetized cats undergoing surgery

**DOI:** 10.5455/javar.2021.h546

**Published:** 2021-11-01

**Authors:** Hirokazu Kamohara, Toshiko Kamohara, Yoshiaki Hikasa

**Affiliations:** 1 Kamohara Animal Hospital, Kochi-shi, Japan; 2 Joint Department of Veterinary Medicine, Faculty of Agriculture, Tottori University, Tottori-shi, Japan

**Keywords:** Cat, isoflurane, ketamine, medetomidine, midazolam, stress hormone

## Abstract

**Objective:**

The purpose of this study was to compare the effects of pretreatment with medetomidine (Me), midazolam (Mi), and ketamine (Ke) on stress-related neurohormonal and metabolic responses in isoflurane-anesthetized cats undergoing ovariohysterectomy and castration.

**Materials and Methods:**

We prospectively recruited 112 client-owned healthy mixed-breed cats. In both surgeries, we divided the cats into seven groups (eight cats per group): non-treatment (control), Me (50 μg/kg), Mi (0.5 mg/kg), Ke (5 mg/kg), Me + Mi, Me + Ke and Me + Mi + Ke administered intramuscularly. After pretreatments, we maintained anesthesia with isoflurane and oxygen. Venous blood was taken before pretreatment, pre- and post-operatively during anesthesia, and at early- and complete-recovery.

**Results:**

Both plasma adrenaline and noradrenaline were reduced during anesthesia in all groups. Plasma cortisol increased during anesthesia and at early recovery in non-Me-treated groups, whereas it decreased in Me-treated groups in both surgeries. Plasma insulin and non-esterified fatty acid (NEFA) decreased, and glucose increased during anesthesia in all groups, but hyperglycemia and decrease in NEFA were greater in Me-treated groups.

**Conclusions:**

In isoflurane-anesthetized cats undergoing surgeries, premedication with Me alone and in combination is useful for reducing the perioperative stress-related increase in cortisol and catecholamines except for hyperglycemia.

## Introduction

The alpha(α)_2_-adrenoceptor agonist, medetomidine (Me), is widely used in feline practice as an excellent analgesic and muscle relaxant. However, it induces undesirable effects like hyperglycemia, hypoinsulinemia, emesis, and bradyarrhythmias in cats [1–6]. A combination of Me with midazolam (Mi) and/or ketamine (Ke) produces good anesthesia in cats, with a reduction of adverse effects or the potentiation of analgesia [7–15].

Stress is defined as an animal’s biological response to a disruption or threat to homeostasis [16]. Animals undergo neurohormonal and metabolic changes in response to stressors such as anxiety, excitement, pain, and anesthesia [17–19]. These changes are characterized by increased levels of cortisol, catecholamines, glucose, and non-esterified fatty acid (NEFA) in the blood and decreased levels of insulin in the blood [17–19]. These events are closely associated with actions mediated by α_2_-adrenoceptors. Me inhibits catecholamine release, insulin secretion, and lipolysis in cats and induces hyperglycemia [2].

On the other hand, premedication with Me has been shown to prevent or delay the stress response induced by ovariohysterectomy in isoflurane-anesthetized dogs [20]. In halothane-anesthetized dogs undergoing ovariohysterectomy, treatment with Me prevents increased plasma cortisol concentrations during the surgery and early-recovery process [21]. Additionally, Me has been reported to have some advantages over acepromazine in terms of reducing perioperative levels of stress-related hormones such as plasma catecholamine and cortisol [22]. However, to the best of our knowledge, there are no published reports on the effects of pretreatment with Me alone and in combination for stress responses in anesthetized cats undergoing surgery. Thus, the purpose of this study was to compare the effects of Me, Mi, and Ke on key stress-related neurohormonal and metabolic variables in isoflurane-anesthetized cats undergoing ovariohysterectomy and castration when used alone or in combination. This study was designed to assess stress responses focusing on clinically important perioperative stages in feline practice.

## Materials and Methods

### Ethical approval

This study utilized client-owned animals and adhered to internationally recognized high standards of individual veterinary clinical patient care (“best practice”). For the procedure described in this study, informed consent was obtained from the owners of all animals described in this study. 

### Animals

We prospectively recruited 112 client-owned mixed-breed cats (56 males and 56 females) at the Kamohara Animal Hospital for ovariohysterectomy or castration. They were clinically healthy and ranged in age from 6 months to 1 year, weighing 3.5 ± 0.8 kg [mean ± standard deviation (SD)]. We obtained informed consent from every cat owner to collect data. Physical and routine hematological examinations before the study revealed that all values were within normal physiological ranges. The owner brought the cat to our hospital early in the morning on the day of surgery. Then, we prepared for surgery and anesthesia. After preparation, each cat was rested in a darkened cage for 2–3 h before anesthesia. After complete recovery from anesthesia, cats were allowed free drinking water and were fed. All cats fasted for 12 h, but the water was available *ad libitum*.

#### Study protocol

We randomly assigned cats to one of seven treatment groups (eight cats in each group) in both ovariohysterectomy and castration. We gave each cat an intramuscular injection of the following pretreatment: physiological saline solution (0.5 ml), 50 μg/kg Me (1 mg/ml), 0.5 mg/kg Mi (5 mg/ml), 5 mg/kg Ke (50 mg/ml), 50 μg/kg Me + 0.5 mg/kg Mi, 50 μg/kg Me + 5 mg/kg Ke and 50 μg/kg Me + 0.5 mg/kg Mi + 5 mg/kg Ke. The groups will be called control, Me, Mi, Ke, MM, MK, and MMK, respectively. For drug combination, we mixed all drugs in a syringe immediately before injection. Twenty minutes (min) after each premedication, anesthesia was induced by an intravenous injection of 5 mg/kg propofol (10 mg/ml) in the control and Mi groups. In other groups, we induced anesthesia with 4% isoflurane in oxygen at a total gas flow rate of 1.5 l/min using a face mask attached to ADS 1000 veterinary anesthesia delivery system (Engler, Hialeah, FL). After induction of anesthesia, we inserted a cuffed endotracheal tube. We placed the cats in the supine position and maintained them at a surgical depth of isoflurane anesthesia through the non-rebreathing system under controlled ventilation. Castration or ovariohysterectomy was performed using standard methods. Preoperatively, all cats received a subcutaneous injection of an analgesic (0.3 mg/kg meloxicam; Inflacam, Chanelle Pharmaceuticals Manufacturing Ltd, Ireland), followed by once daily for several days following surgery, if necessary. Lactated Ringer’s solution was infused intravenously at 10 ml/kg/h during anesthesia. Inhalation of isoflurane was completely stopped approximately 5 min after the end of each operation, and the endotracheal tube was extubated after the laryngeal reflex was observed. During the recovery process, cats were kept in separate cages in a room with the air temperature set at 25°C. General postoperative management and care were performed in all cats. Specific attention to arousal behavior was paid to animals of the control group. After complete recovery, another analgesic, butorphanol (0.1–0.4 mg/kg; Vetorphale, Meiji Seika, Tokyo, Japan), was injected intramuscularly, especially in control, to cats with signs of pain such as vocalization, anorexia, and posture. No life-threatening events in surgery, anesthesia, and postoperative management occurred in all cats.

#### Anesthesia and intraoperative monitoring

We used an agent-specific precision vaporizer to administer isoflurane. We drew gas samples from the breathing circuit through a tube attached to an adapter positioned at the oral end of an endotracheal tube. We measured expired end-tidal isoflurane (EtIso) and carbon dioxide (EtCO_2_) concentrations, arterial oxygen saturation of pulse oximetry (SpO_2_), heart rate (HR), respiration rate (RR), rectal temperature, and mean blood pressure (MBP) using the oscillometric method continuously or intermittently using a multi-parameter monitor (BSM-5192; Nihon Kohden, Tokyo, Japan) during anesthesia. During controlled ventilation, RR was adjusted to a range of approximately 25–35 mmHg EtCO_2_. The mean duration of anesthesia in castrated and ovariohysterectomized groups ranged from 24.8–31.3 to 48.1–60.0 mins, respectively. SpO_2_ was >98% in all cats. The mean RR in castrated and ovariohysterectomized groups ranged from 11.3–14.4 to 9.3–11.1 breaths/min, respectively. The mean rectal temperature during surgery ranged from 37.8°C to 36.8°C in castrated groups, and from 36.9°C to 35.5°C in ovariohysterectomized groups. There were no significant differences in duration of anesthesia, RR, EtCO_2_, and SpO_2_, and rectal temperature values across groups in both surgeries.

#### Scoring of behavioral changes during recovery

We assessed the overall quality of recovery from anesthesia using the previously published scoring methods [23] as follows: score 1 = excellent; score 2 = good; score 3 = moderate; score 4 = poor; score 5 = extremely poor. The observer was blinded to each treatment.

#### Blood sample collection

Blood sample collection was focused on clinically important perioperative stages. Blood samples (2 ml) were collected from a 24-gauge catheter inserted into the cephalic vein five times; immediately before pretreatment (baseline), 5 min after induction of anesthesia and before the surgical procedure during anesthesia (preoperatively), after completing the surgical procedure during anesthesia (post-operatively), at head-up motion after the removal of the tracheal tube after discontinuation of anesthesia (early-recovery), and at recovery to normal behavior similar to that before pretreatment (complete-recovery). Blood sampling time to post-operation, early- and complete-recovery is listed in Table 1. There were no significant differences in blood sampling times to post-operation and complete-recovery across groups in both surgeries. 

#### Sample processing and analysis

To prevent clotting, blood was mixed with ethylenediaminetetraacetic acid. Centrifuge the samples immediately; the plasma was separated and frozen at −76°C until analysis. We determined the glucose, NEFA, insulin, cortisol, adrenaline, and noradrenaline levels using previously published methods [2,23]. The glucose and NEFA concentrations were determined using an enzyme assay and a spectrophotometer, respectively. Insulin and cortisol levels were determined using a solid phase two-site enzyme immunoassay and a solid phase antibody radioimmunoassay. Catecholamines were isolated and quantified using high-performance liquid chromatography and an electrochemical detector.

#### Statistical analysis

We analyzed all data obtained using statistical software (Prism 7.0; GraphPad, CA). Data are presented as the mean ± SD. We tested all data except for score data for normality using the Shapiro–Wilk test. The repeated measures of one-way analysis of variance (ANOVA) were used to examine the differences for changes in variables within each group. The post hoc Dunnett’s multiple comparisons test was used to identify differences from baseline within the group. One-way ANOVA and post hoc Tukey’s multiple comparisons test were used to determine differences among the groups. In both tests, the significance level was set at p < 0.05. We analyzed score data using the Wilcoxon–Mann–Whitney test for treatment comparisons, and a p < 0.00714 was considered significant by Bonferroni correction.

### Results

#### EtIso, HR and MBP during anesthesia

**Figure 1.**
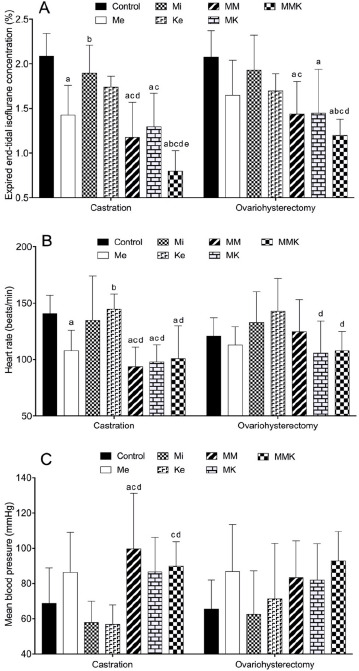
Mean expired EtIso concentration (A), HR (B) and MBP (C) during isoflurane anesthesia and surgery in cats premedicated with Me (50 μg/kg), Mi (0.5 mg/kg), and Ke (5 mg/kg), either alone or in combination. Each vertical bar indicates the mean and SD of eight cats. MM = Me and Ke, MK = Me and Ke; MMK = Me, Mi, and Ke; ^a^ significantly different from control; ^b^ significantly different from Me; ^c^ significantly different from Mi; ^d^ significantly different from Ke; ^e^ significantly different from MK; the significance level is p < 0.05.

In both surgeries, the mean EtIso concentration was significantly lower in Me and Me-combined groups (1.43% ± 0.33%, 1.18% ± 0.39%, 1.30% ± 0.37% and 0.80% ± 0.23% in castration and 1.65% ± 0.39%, 1.44% ± 0.36%, 1.45% ± 0.49% and 1.20% ± 0.18% in ovariohysterectomy of Me, MM, MK and MMK group, respectively) than in the control group (2.09% ± 0.25% and 2.08% ± 0.29%) and was lowest in the MMK group (0.80% ± 0.23% and 1.20% ± 0.18%). The mean HR was significantly lower in Me-treated groups than in non-Me groups in either castrated or ovariohysterectomized cats. The mean MBP was significantly higher in MM and MMK groups than in non-Me-treated groups in castrated cats. The results are shown in Figure 1.

**Table 1.** Blood sampling time to post-operation, early-recovery, and complete-recovery phases in isoflurane-anesthetized cats premedicated with medetomidine (Me; 50 μg/kg), midazolam (Mi; 0.5 mg/kg) and ketamine (Ke; 5 mg/kg), either alone or in combination.

Surgery	Group	During anesthesia (mins)	After discontinuing anesthesia (mins)	
		Post-operation	Early-recovery	Complete-recovery
Castration	Control	21.5 ± 2.6	17.8 ± 15.7	323 ± 29
	Me	23.3 ± 5.4	25.0 ± 9.7	323 ± 46
	Mi	27.9 ± 8.9	14.8 ± 5.0	342 ± 44
	Ke	21.0 ± 3.3	16.0 ± 14.1	331 ± 54
	MM	23.1 ± 7.6	35.0 ± 18.9	301 ± 45
	MK	31.3 ± 9.6	32.0 ± 12.9	324 ± 32
	MMK	30.6 ± 5.6	43.3 ± 23.9	335 ± 37
OHE	Control	48.0 ± 7.3	15.0 ± 7.0	312 ± 34
	Me	50.9 ± 4.0	34.0 ± 30.4	338 ± 17
	Mi	52.5 ± 6.1	19.3 ± 7.3	308 ± 27
	Ke	46.8 ± 4.1	14.1 ± 4.3	295 ± 23
	MM	58.8 ± 11.0	39.0 ± 19.1	307 ± 22
	MK	60.0 ± 7.8	30.0 ± 9.6	312 ± 21
	MMK	54.3 ± 14.2	47.6 ± 35.0	312 ± 30

Values represent mean ± SD of eight cats. OHE = Ovariohysterectomy; MM = Me and Ke; MK = Me and Ke; MMK = Me, Mi and Ke.

#### Adrenaline

**Figure 2.**
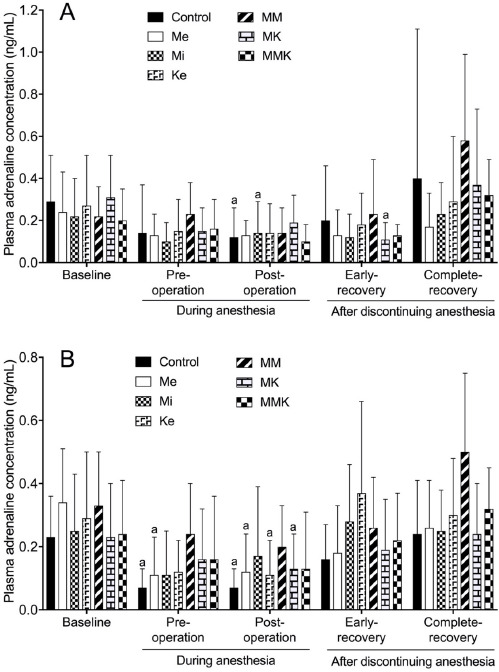
Plasma adrenaline concentration during isoflurane anesthesia and recovery phase in castrated (A) or ovariohysterectomized (B) cats premedicated with Me (50 μg/kg), Mi (0.5 mg/kg), and Ke (5 mg/kg), either alone or in combination. Each vertical bar indicates the mean and SD of eight cats. MM = Me and Ke, MK = Me and Ke; MMK = Me, Mi, and Ke; ^a^ significantly different from baselinehe significance level is *p *< 0.05.

The effects of pretreatments with Me, Mi, Ke alone and in combinations on the changes of adrenaline concentration are shown in Figure 2. In castrated cats, adrenaline concentration in all groups decreased significantly or tended to decrease pre- and post-operatively compared with the baseline values (ranged 0.20 ± 0.15 to 0.31 ± 0.20 ng/ml). A significant decrease in adrenaline concentration was also observed at early-recovery in MK group. In ovariohysterectomized cats, adrenaline concentration decreased significantly or tended to decrease pre- and post-operatively compared with baseline in all groups. Adrenaline concentration during anesthesia and early-recovery (0.23 ± 0.15, 0.14 ± 0.12 and 0.23 ± 0.26 ng/ml in castration; 0.24 ± 0.16, 0.20 ± 0.13 and 0.26 ± 0.16 ng/ml in ovariohysterectomy) in the MM group did not significantly change from the baseline (0.22 ± 0.14; 0.33 ± 0.17 ng/ml) in both surgeries.

#### Noradrenaline

**Figure 3.**
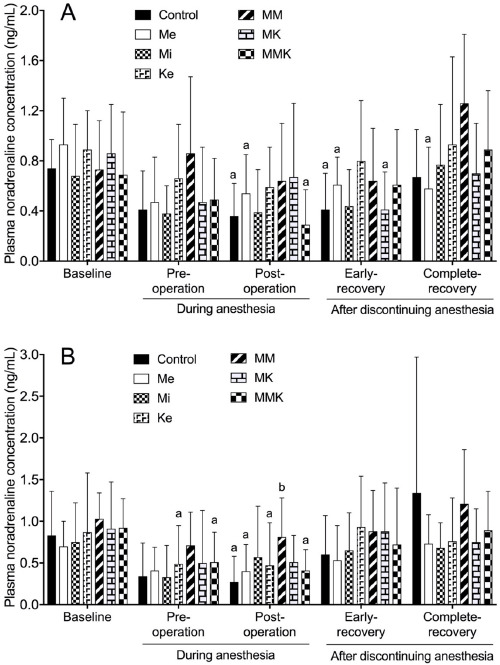
Plasma noradrenaline concentration during isoflurane anesthesia and recovery phase in castrated (A) or ovariohysterectomized (B) cats premedicated with Me (50 μg/kg), Mi (0.5 mg/kg), and Ke (5 mg/kg), either alone or in combination. Each vertical bar indicates the mean and SD of eight cats. MM = Me and Ke; MK = Me and Ke; MMK = Me, Mi, and Ke; ^a^ significantly different from baseline; ^b^ significantly different from control; the significance level is *p *< 0.05.

The effects of pretreatments with Me, Mi, Ke alone and in combinations on the changes of noradrenaline concentration are shown in Figure 3. In castrated cats, noradrenaline concentration in control, Me, MK and MMK groups decreased significantly at pre- and post-operation and/or early-recovery compared with baseline, but that in Mi, Ke, and MM groups did not significantly change at any phase compared with baseline (0.68 ± 0.41, 0.89 ± 0.31 and 0.73 ± 0.39 ng/ml). In ovariohysterectomized cats, noradrenaline concentration decreased significantly pre- and/or post-operatively compared with baseline in control, Me, Ke, and MMK groups. Postoperative noradrenaline concentration was significantly higher in the MM group (0.81 ± 0.47 ng/ml) than in the control group (0.27 ± 0.31 ng/ml).

#### Cortisol

**Figure 4.**
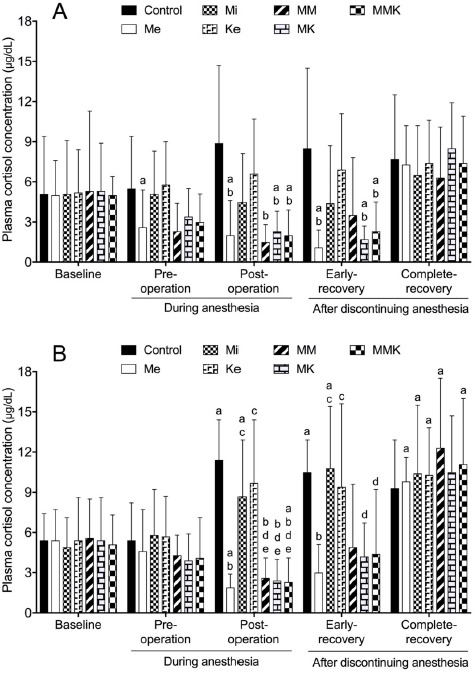
Plasma cortisol concentration during isoflurane anesthesia and recovery phase in castrated (A) or ovariohysterectomized (B) cats premedicated with Me (50 μg/kg), Mi (0.5 mg/kg) and Ke (5 mg/kg), either alone or in combination. Each vertical bar indicates the mean and SD of eight cats. MM = Me and Ke; MK = Me and Ke; MMK = Me, Mi, and Ke; ^a^ significantly different from baseline; ^b^ significantly different from control; ^c^ significantly different from Me; ^d^ significantly different from Mi; ^e^ significantly different from Ke; the significance level is *p *< 0.05.

The effects of pretreatments with Me, Mi, Ke alone and in combinations on the changes of cortisol concentration are shown in Figure 4. In castrated cats, cortisol concentration in the control group did not significantly change during anesthesia and recovery compared with baseline, whereas that in the Me, MK, and MMK groups decreased significantly pre- and post-operatively and at early-recovery compared with baseline. Cortisol concentration postoperatively and/or at early-recovery was significantly lower in Me-treated groups (Me, 2.0 ± 2.6 μg/dl; MM, 1.5 ± 1.3 μg/dl; MK, 2.3 ± 1.5 μg/dl; and MMK, 2.0 ± 1.9 μg/dl at post-operation) than in the control group (8.9 ± 5.8 μg/dl at post-operation). In ovariohysterectomized cats, cortisol concentration in control and Mi groups increased significantly post-operatively and at early- and/or complete-recovery compared with baseline, whereas that in the Me and MMK groups decreased significantly post-operatively. Cortisol concentration post-operatively and/or at early-recovery was significantly lower in Me-treated groups than in non-Me groups. Cortisol concentration post-operatively and at the recovery phase tended to be greater in ovariohysterectomy than castration in each group. A significant difference (p < 0.05) in cortisol concentration between surgeries was observed at early-recovery and complete-recovery in the Mi and MM groups, respectively (4.4 ± 4.3 μg/dl in castration and 10.8 ± 4.6 μg/dl in ovariohysterectomy of Mi group; 6.3 ± 3.8 μg/dl in castration and 12.3 ± 5.2 μg/dl in ovariohysterectomy of MM group).

#### Insulin

**Figure 5.**
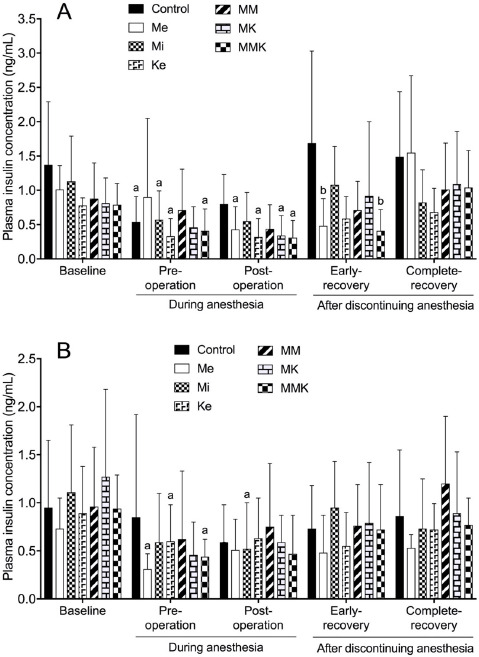
Plasma insulin concentration (ng/ml) during isoflurane anesthesia and recovery phase in castrated (A) or ovariohysterectomized (B) cats premedicated with Me (50 μg/kg), Mi (0.5 mg/kg) and Ke (5 mg/kg), either alone or in combination. Each vertical bar indicates the mean and SD of eight cats. MM = Me and Ke; MK = Me and Ke; MMK = Me, Mi, and Ke; ^a^ significantly different from baseline; ^b^ significantly different from control; the significance level is *p *< 0.05.

The effects of pretreatments with Me, Mi, Ke alone and in combinations on the changes of insulin concentration are shown in Figure 5. In castrated cats, insulin concentration in all groups except for MM group decreased significantly pre- and/or postoperatively (0.54 ± 0.37, 0.32 ± 0.27, 0.43 ± 0.33, 0.57 ± 0.42, 0.32 ± 0.27, 0.34 ± 0.29 and 0.31 ± 0.25 ng/ml in control, Me, Mi, Ke, MK and MMK, respectively) compared with baseline (ranged 0.78 ± 0.11 to 1.37 ± 0.92 ng/ml). Insulin concentration in the Me and MMK groups was also significantly lower in early-recovery compared with baseline. Insulin concentration at early-recovery was significantly lower in the Me and MMK groups (0.48 ± 0.40 and 0.41 ± 0.31 ng/ml) than in the control group (1.69 ± 1.34 ng/ml). In ovariohysterectomized cats, insulin concentration in the Me, Mi, Ke, and MMK groups decreased significantly pre- and/or post-operatively compared with baseline. Insulin concentration in the control, MM and MK groups (0.59 ± 0.39, 0.62 ± 0.71, and 0.46 ± 0.34 ng/ml) tended to decrease insignificantly pre- and/or postoperatively compared with baseline (0.95 ± 0.70, 0.96 ± 0.62, and 1.27 ± 0.91 ng/ml).

#### Glucose

**Figure 6.**
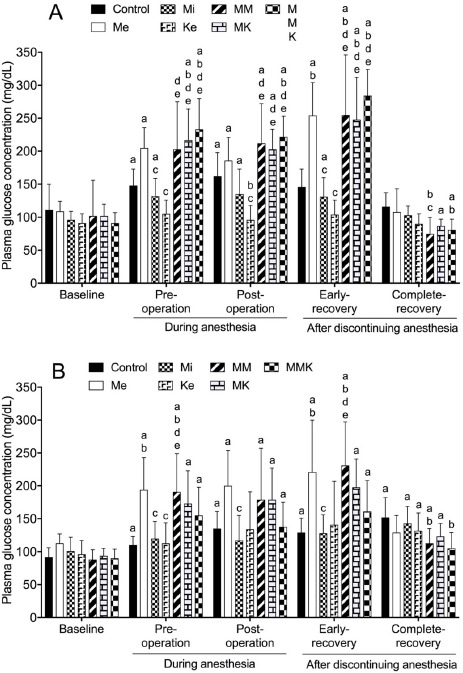
Plasma glucose concentration during isoflurane anesthesia and recovery phase in castrated (A) or ovariohysterectomized (B) cats premedicated with Me (50 μg/kg), Mi (0.5 mg/kg) and Ke (5 mg/kg), either alone or in combination. Each vertical bar indicates the mean and SD of eight cats. MM = Me and Ke; MK = Me and Ke; MMK = Me, Mi, and Ke; ^a^ significantly different from baseline; ^b^ significantly different from control; ^c^ significantly different from Me; ^d^ significantly different from Mi; ^e^ significantly different from Ke; the significance level is *p *< 0.05.

The effects of pretreatments with Me, Mi, Ke alone and in combinations on glucose concentration changes are shown in Figure 6. In castrated cats, glucose concentration in all groups except for the Ke group increased significantly pre- and post-operatively and/or at early-recovery (162 ± 36, 186 ± 35, 135 ± 38, 212 ± 60, 203 ± 30, and 222 ± 31 mg/dl in control, Me, Mi, MM, MK and MMK at post-operation, respectively) compared with baseline (ranged 96 ± 13 to 111 ± 39 mg/dl). Glucose concentration pre- and post-operatively and during early-recovery were significantly higher in the Me-treated groups than in non-Me groups. In ovariohysterectomized cats, glucose concentration increased significantly pre- and post-operatively and at early- and/or complete-recovery compared with baseline in all groups. Glucose concentration pre- and post-operatively and/or during early-recovery were significantly higher in Me-treated groups than in non-Me groups.

#### Non-esterified fatty acid 

**Figure 7.**
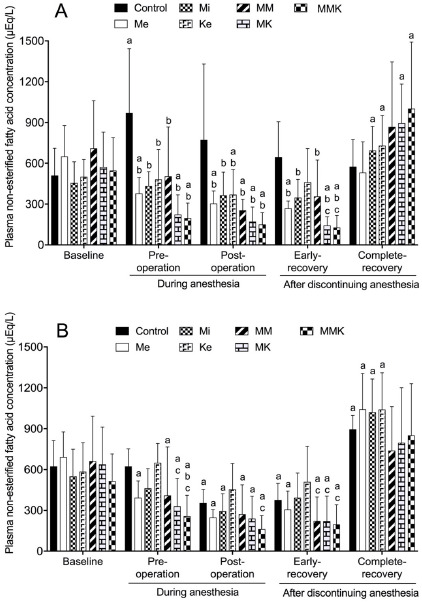
Plasma non-esterified fatty acid concentration during isoflurane anesthesia and recovery phase in castrated (A) or ovariohysterectomized (B) cats premedicated with Me (50 μg/kg), Mi (0.5 mg/kg) and Ke (5 mg/kg), either alone or in combination. Each vertical bar indicates the mean and SD of eight cats. MM = Me and Ke; MK = Me and Ke; MMK = Me, Mi, and Ke; ^a^ significantly different from baseline; ^b^ significantly different from control; ^c^ significantly different from Ke; the significance level is *p *< 0.05.

The effects of pretreatments with Me, Mi, Ke alone and in combinations on the changes of NEFA concentration are shown in Figure 7. In castrated cats, NEFA concentration in the control group increased significantly preoperatively compared with baseline, whereas that in Me-treated groups decreased significantly pre- and post-operatively and/or during early recovery. NEFA concentration pre- and post-operatively and at early-recovery were significantly lower in the Me, Mi, Ke, and Me-combined groups than in the control group. NEFA concentration in all groups except for the Ke group decreased significantly pre- and post-operatively and/or at early-recovery compared with baseline in ovariohysterectomized cats. NEFA concentration pre- and post-operatively and/or during early-recovery were significantly or insignificantly lower in Me-treated groups than in non-Me groups.

#### Recovery score

The results on the scoring of behavioral changes during recovery are shown in Table 2. In both castrated and ovariohysterectomized cats, score data were significantly lower in Me, MK, and MMK groups than in non-Me groups. No significant difference in score among Me-treated groups was observed, except that it was lower in the MMK group than in the MM group.

### Discussion

The present results revealed that adrenaline and noradrenaline concentrations decreased during isoflurane anesthesia in cats undergoing castration or ovariohysterectomy. It has been reported that isoflurane inhibits the secretion of catecholamines in bovine adrenal chromaffin cells at concentrations within the range encountered during general anesthesia [24], showing that isoflurane anesthesia itself inhibits the release of catecholamines due to suppressing the sympathetic-adrenomedullary activity in cats. As Me is known to decrease plasma adrenaline and noradrenaline concentrations in cats [2], we postulated that Me treatment might significantly reduce plasma catecholamine levels during isoflurane anesthesia. However, the present results (Figs. 2 and 3) demonstrated that Me treatment did not enhance the inhibition of catecholamine release compared to that in the control group during isoflurane anesthesia. This may be responsible for the decrease in isoflurane concentration by Me treatment. The present results also revealed that catecholamine concentrations during isoflurane anesthesia were higher in the MM group than in the control group in ovariohysterectomized cats (Figs. 2 and 3). Plasma adrenaline and noradrenaline concentrations are increased by Mi in cats [25] and by Ke in dogs [26]. Ke-induced increases in plasma adrenaline and noradrenaline are mitigated by Mi [27] and even abolished by Me [26]. Therefore, both the reduction of isoflurane concentration by MM treatment and the effect of Mi on catecholamine release may be involved in preventing an excessive decrease in catecholamine concentrations during isoflurane anesthesia.

Previous studies have shown that Me alone or MM does not significantly affect plasma cortisol concentration in healthy cats without inhalant anesthesia and surgery [2,25]. In the present study, plasma cortisol concentration increased significantly in the control and non-Me groups post-operatively and at the early-recovery phase in isoflurane-anesthetized cats, whereas it decreased in Me-treated groups (Fig. 4). These results in cats were similar to previous reports in dogs, which showed that Me premedication reduced or delayed the increase in cortisol concentrations induced by ovariohysterectomy [20,21]. Ke alone increased cortisol concentration from sympathomimetic effects in dogs [26] and humans [28] and did not prevent further cortisol release from surgery in humans [29]. The present study also confirmed that Ke premedication did not diminish the increase in cortisol concentration during isoflurane anesthesia and surgery (Fig. 4). Furthermore, cortisol concentration post-operatively and during the recovery phase tended to be greater in ovariohysterectomy than in castration in each group, supporting that cortisol release depends on the type of surgery, nociceptive stimulation, and the degree of trauma [17]. Overall, the present findings indicate that pretreatment with Me alone and in combination is useful to suppress an excessive adrenocortical activity perioperatively in isoflurane-anesthetized cats. In the present study, the quality of recovery from anesthesia was better in Me-treated groups than in non-Me groups (Table 2), supporting that Me pretreatment reduces the adrenocortical response during the arousal from isoflurane anesthesia and surgery in cats.

**Table 2.** Behavioral recovery score after castration and ovariohysterectomy in isoflurane-anesthetized cats premedicated with Me (50 μg/kg), Mi (0.5 mg/kg), and Ke (5 mg/kg), either alone or in combination.

Group	Castration	Ovariohysterectomy
Control	4 (3–5)	4 (3–5)
Me	2 (1–3)^a^	2 (1–4)^a^
Mi	4 (3–5)^b^	4 (3–5)^b^
Ke	4 (3–4)^b^	4 (3–4)^b^
MM	3 (2–4)	3 (2–4)
MK	2 (2–3)^acd^	2 (2–3)^acd^
MMK	2 (1–3)^acd^	2 (1–3)^acde^

Values represent the median (range) of eight cats. MM = Me and Ke; MK = Me and Ke; MMK = Me, Mi, and Ke.

a Significantly different from control.

b Significantly different from Me.

c Significantly different from Mi.

d Significantly different from Ke.

e Significantly different from MM.

The significance level is *p *< 0.00714.

Isoflurane itself inhibits insulin release from pancreatic α-cells and glucose tolerance independent of a dosage up to 1.5 minimal alveolar concentration in humans [30]. Anesthesia with low- or high-dose isoflurane reduces plasma insulin levels in rats [31]. In the current study, the insulin concentration in the control group decreased during isoflurane anesthesia in either ovariohysterectomy or castration, supporting the hypothesis that isoflurane anesthesia can inhibit insulin release in cats as well as in humans. Me inhibits insulin secretion via α2-adrenoceptors on α-cells of the pancreas [32]. In previous studies, an intramuscular Me at a wide range of dosages decreased plasma insulin concentration in unanesthetized cats [2]. Mi alone did not significantly alter plasma insulin levels in healthy cats [25]. Ke alone did not affect basal plasma insulin concentration at the initial stage of cesarean section in humans [29]. In the current study, the decrease in plasma insulin concentration tended to be greater in Me-treated groups than in the control group during isoflurane anesthesia and at early-recovery, suggesting that Me premedication enhances the inhibition of insulin release in isoflurane anesthesia and post-operatively in cats.

The present results revealed that blood glucose increased during isoflurane anesthesia and recovery in the control group. This increase in blood glucose may be responsible for several factors, including surgical injuries, increased cortisol, decreased insulin, and decreased peripheral use of glucose associated with inhalant anesthesia [17]. Me induces dose-dependent hyperglycemia with inhibition of insulin release in healthy cats [2]. In the present study, glucose concentration during anesthesia and early-recovery was higher in Me-treated groups than in non-Me groups, showing that Me premedication facilitates the hyperglycemia during isoflurane anesthesia and surgery in cats. This enhancement of hyperglycemia by Me may be mainly due to Me-induced inhibition of insulin release via α2-adrenoceptors on pancreatic α-cells [32].

Conversely, an intravenous administration of 10 μg/kg dexmedetomidine induced hyperglycemia but did not significantly alter insulin concentration in healthy cats [6]. In the present study, the degree of hyperglycemia in Me-treated groups seemed not to be correlated with decreased insulin levels, suggesting that insulin is not the only factor affecting glucose levels in Me-treated cats. Nevertheless, this hyperglycemia may limit the use of Me in cats with metabolic and neurohormonal problems such as diabetes mellitus, ketosis, and glycosuria. To overcome Me-induced hyperglycemia and possible risks, it may be necessary to consider the early use of antagonists after surgery is completed.

Because it is influenced by hormones such as cortisol and catecholamines, the change in NEFA concentration is significant as a metabolic index of the stress response [17]. Cortisol and catecholamines stimulate lipolytic activity, while insulin inhibits it [17]. In the present study, NEFA concentration in the control group increased slightly preoperatively in castrated cats but decreased post-operatively and during early recovery in ovariohysterectomized cats. These results may be attributed to the complicated effects of decreased insulin, decreased catecholamine and increased cortisol in isoflurane anesthesia and surgery. Me reduces plasma NEFA concentration; however, Mi does not reduce it in unanesthetized cats [2,25]. Ke alone does not significantly alter NEFA concentrations in dogs [26]. In the present study, treatment with Me alone and in combination significantly reduced NEFA concentration during anesthesia and early recovery in cats. These results may be due to the suppression of lipolytic activity via α2-adrenoceptors on adipose tissues as well as decreased cortisol and catecholamine concentrations [17,32]. Although the fluctuation of NEFA concentration may not be directly harmful to anesthetized cats, this change may indirectly reflect the sympathetic-adrenal activation associated with anesthesia and surgery. Therefore, the Me pretreatment-induced reduced NEFA concentration may be clinically significant as a metabolic index of inhibition of sympathetic-adrenal activation in isoflurane-anesthetized cats undergoing surgery.

### Conclusion

Isoflurane anesthesia itself inhibited decreased adrenaline and noradrenaline concentrations but increased cortisol concentrations and hyperglycemia in castrated and ovariohysterectomized cats. Pretreatment with Me alone and in combination reduced cortisol release during isoflurane anesthesia and in the early-recovery phase as well as the improvement of the quality of recovery. No remarkable differences in sympathetic-adrenal and metabolic responses were observed between Me-treated groups, except that MM treatment prevented an excessive decrease in catecholamine concentrations during isoflurane anesthesia. This study demonstrated newly that pretreatments with Me alone and in combination are useful for the prevention of stress responses induced by isoflurane anesthesia and surgery in feline practice.

### List of abbreviations

ANOVA: One-way analysis of variance; EtCO_2_: Expired end-tidal carbon dioxide; EtIso: Expired end-tidal isoflurane; HR: Heart rate; Ke: Ketamine; MBP: Mean blood pressure; Me: Medetomidine; Mi: Midazolam; MK: Medetomidine and ketamine; MM: Medetomidine and midazolam; MMK: Medetomidine, midazolam and ketamine; NEFA: non-esterified fatty acid; RR: Respiration rate; SpO_2_: Arterial oxygen saturation of pulse oximetry.
